# Myositis ossificans of the serratus anterior as a rare complication of massage: a case report

**DOI:** 10.1186/s13256-015-0628-2

**Published:** 2015-06-16

**Authors:** Jian Wei, Yingwei Jia, Bingsheng Liang

**Affiliations:** Department of Orthopedics, The Second Hospital, Shanxi Medical University, Taiyuan, 030001 China

**Keywords:** Myositis ossificans, Nape massage, Numbness, Serratus anterior

## Abstract

**Introduction:**

Myositis ossificans usually occurs in the vicinity of the elbow, knee joints, or hip joints, following obvious trauma or surgery. This is the first report on myositis ossificans of the serratus anterior.

**Case presentation:**

In this report we present a case of myositis ossificans within the serratus anterior which developed as a complication due to long-term nape massage. The patient was a 29-year-old Han woman. Because heterotopic ossificans constricted her brachial plexus the surface of her right upper arm was slightly numb; the symptom disappeared after surgery.

**Conclusion:**

This case highlights that myositis ossificans can occur in the serratus anterior following long-term nape massage.

## Introduction

Myositis ossificans is a heterotopic bone formation within a muscle. It is mainly found in the muscle of extremities, but myositis ossificans of the serratus anterior has never previously been reported. The current report presents the case of a 29-year-old woman with a very rare form of myositis ossificans of the serratus anterior developed due to long-term nape massage.

## Case presentation

**Fig. 1:**
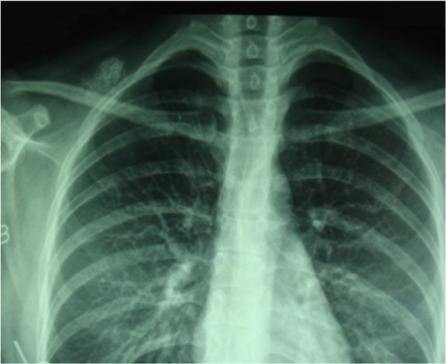
Chest X-ray demonstrating a mass with a high density shadow at the superior and posterior right clavicle

**Fig. 2:**
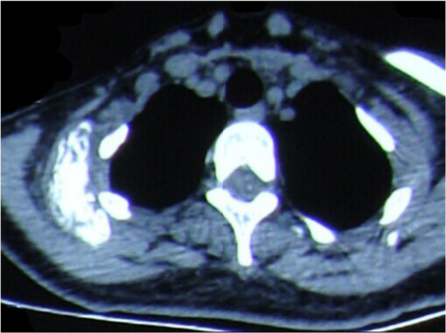
Axial computed tomography scan image showing a mixed density shadow in the serratus anterior

**Fig. 3:**
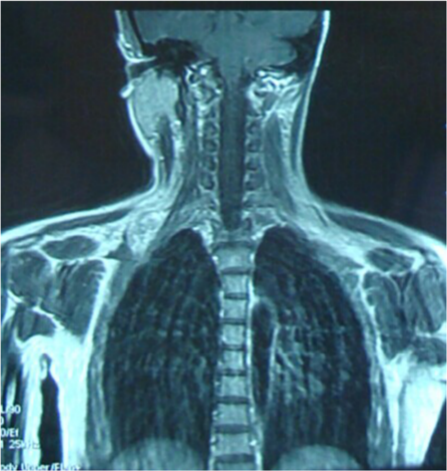
Coronal magnetic resonance imaging scan showing abnormal signal in the serratus anterior at the right-side of the chest wall

**Fig. 4:**
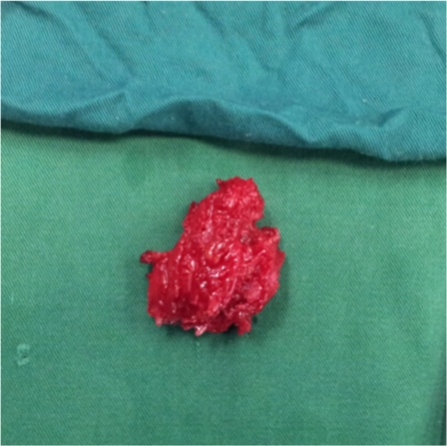
The resected tumor

**Fig. 5:**
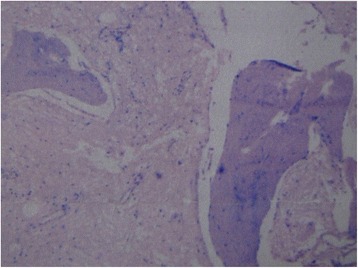
Histopathological examination showing lesions consistent with myositis ossificans. ×400

A 29-year-old Han woman presented with a chief complaint of a tumor under her right clavicle for 5 months, and right shoulder soreness and numbness of the exterior of her right upper arm for 10 days. At physical examination there was a red and warm mass over her right shoulder with mild pain and tenderness. The mass was tough, hardly moveable, and base-fixed. The strength of her right upper limb muscles was 4+. A chest X-ray showed a mass with a high density shadow at the superior posterior right clavicle (Fig. 1). A right shoulder computed tomography scan showed a mixed density shadow in the serratus anterior, which correlated with clinical findings (Fig. 2). A neck magnetic resonance imaging scan showed abnormal findings in the serratus anterior at the right-side of her chest wall, which also correlated clinically (Fig. 3). An electromyogram showed a few positive sharp waves in muscles innervated by the brachial plexus upper trunk. Motor unit potential mixing interference patterns, compound muscle action potential, motor nerve conduction velocity, and sensory nerve conduction velocity were within the normal range. Her right brachial plexus upper trunk was slightly damaged. Based on the above examinations, the patient’s condition was diagnosed as a tumor beneath the right clavicle. She underwent surgery as a treatment. During the operation, the tumor boundary was found to be on the posterolateral side of the middle scalene; anteroinferior of the trapezius, levator scapulae, and postscalene; and behind the brachial plexus cervical nerves 5 and 6 (C5–6). The tumor invaded the serratus anterior. Anteromedial tumor adhered to the C5–6 nerve branches, posterolaterally adhered to the suprascapular nerve, and adhered to the rear edge of the brachial plexus sheath and omohyoid in the front. Under a microscope, the neurovascular and protective brachial plexus sheath, and the C5–6 nerve branches and the suprascapular nerve were carefully separated; tumor subordinates back below the upper edge of the scapula notch were detected; and the tumor of 7×4×3cm size was completely resected (Fig. 4). Intraoperative suprascapular nerve stimulation and muscle contractions were normal; the brachial plexus upper trunk was able to be stimulated; contraction of deltoid, biceps, and flexor carpi were normal; and stimulation of the dorsal scapular nerve, scapular muscles, and rhomboid muscle revealed normal contraction. No intraoperative frozen section was obtained. A postoperative histopathological examination showed that lesions were consistent with myositis ossificans (Fig. 5). The soreness of her right shoulder and right arm numbness disappeared completely postoperatively. After 1 year, she did not have any abnormalities.

## Discussion

Myositis ossificans is characterized by abnormal heterotopic ossification formation, typically involving muscles, tendons, ligaments, fascia, and aponeurosis. It can be divided into three subtypes: traumatic myositis ossificans, nervous myositis ossificans, and progressiva myositis ossificans. Progressiva myositis ossificans is a rare, hereditary, progressive connective tissue disorder, characterized by congenital malformation of the great toes; progressive heterotopic ossification occurs mainly in the neck, chest and back [1]. This patient’s thumbs and toes were normal, her spine was midline, and the results of blood tests showed no abnormality; hence, progressiva myositis ossificans was ruled out. Nerve myositis ossificans usually occurs after injury to the brain or spinal cord [2]. In this case, the patient did not have any brain or spinal cord injury. Traumatic myositis ossificans is secondary to trauma, surgery, or inflammation of a heterotopic ossification; it is a common complication of bone and joint diseases [3]. Hence, the cause of this patient’s reported condition might be due to a long-term (2 years) aggressive nape massage, which could have led to repetitive powerful manipulation injury to the serratus anterior below the collarbone and repetitive muscle bleeding and inflammation forming the adhesions and ossification gradually.

Treatment is usually conservative with analgesics and physical therapy, and excision is considered when excessive pain, joint limitation, or nerve compression is present. Surgery is generally chosen when myositis ossificans is ripe, identified by a higher bone density in X-ray findings and normal red blood cell sedimentation rate and alkaline phosphatase [4]. For our patient, two of the above indicators were in the normal range, her X-ray showed a high bone density, and there were nerve compression symptoms. These findings indicated that surgery was appropriate.

## Conclusions

This is the first case report on myositis ossificans within the serratus anterior, and it suggests that myositis ossificans can occur in the serratus anterior following long-term nape massage. So people who like massages should be advised to refuse rough massage and to question long-term massage that gives them pain.

## Consent

Written informed consent was obtained from the patient for publication of this case report and accompanying images. A copy of the written consent is available for review by the Editor-in-Chief of this journal.

## Abbreviation

C5–6: Cervical nerves 5 and 6
